# Data on CO_2_, temperature and air humidity records in Spanish classrooms during the reopening of schools in the COVID-19 pandemic

**DOI:** 10.1016/j.dib.2021.107489

**Published:** 2021-10-17

**Authors:** Sergio Trilles, Pablo Juan, Somnath Chaudhuri, Ana Belen Vicente Fortea

**Affiliations:** a Institute of New Imaging Technologies, Universitat Jaume I, Av. Vicente Sos Baynat s/n, Castelló de la Plana, Spain; b IMAC, Universitat Jaume I, Av. Vicente Sos Baynat s/n, Castelló de la Plana, Spain; c GRECS, Universitat de Girona, C/ de la Universitat de Girona 10, Girona, Spain; d CIBER of Epidemiology and Public Health (CIBERESP), Madrid, Spain; e Department of Agricultural and Environmental Sciences, Universitat Jaume I, Av. Vicente Sos Baynat s/n, Castelló de la Plana, Spain

**Keywords:** CO_2_, COVID-19, Primary school, Internet of Things, low-cost sensors

## Abstract

In order to reduce the advance of the pandemic produced by COVID-19, many actions and restrictions have been applied and the field of education has been no exception. In Spain, during the academic year 2020–2021, face-to-face teaching generally continued in both primary and secondary schools. Throughout the year, different measures have been taken to reduce the likelihood of contagion in classrooms, one of which was to improve ventilation by opening windows and doors. One of the most commonly used techniques to check for good ventilation has been CO_2_ monitoring. This work provides a set of 80,000 CO_2_ concentration records collected by low-cost Internet of Things nodes, primarily located within twelve classrooms in two primary schools. The published observations were collected between 1 May 2020 and 23 June 2021. Additionally, the same dataset includes temperature, air humidity and battery level observations.

## Specifications Table

**Table:** 

Subject	Computer Networks and Communications, Engineering.
Specific subject area	Application of computing networks and engineering to monitoring environmental phenomena in classrooms using IoT nodes.
Type of data	Text files (Comma Separated Values).
How data were acquired	Data were captured using CO_2_ low-cost sensors.
Data format	Raw sensor data.
Parameters for data collection	The low-cost nodes were deployed in twelve classrooms in two primary schools.
Description of data collection	The dataset was collected from 3 May 2021 to 23 June 2021. This dataset contains CO_2_ concentration, temperature and air humidity. Battery level values are also included.
Data source location	The two schools are located in two small towns in the province of Castellón (Spain).
Data accessibility	Zenodo 10.5281/zenodo.5036228 https://doi.org/10.5281/zenodo.5036228

## Value of the Data

• The dataset presented in this paper can be used for experiments to analyse spatiotemporal variation and air quality dynamics in indoor scenarios.
• Creation of geostatistical models to analyse the relationship of carbon dioxide levels with other environmental or meteorological covariates such as temperature or humidity.
• Development of spatiotemporal risk maps of carbon dioxide concentrations in indoor public places.
• The near-real-time monitoring of carbon dioxide concentration data can help the administration and researchers plan strategies and decide on the adequate ventilation of indoor public places. It can help the school administration plan breaks (and their duration) between regular class schedules to improve students' classroom performance.

## 1 Data Description

The dataset has been published online in the Zenodo data repository [1]. The data presented were collected between 1 May 2021 and 23 June 2021 using a low-cost CO_2_ sensor called SCD30 (https://bit.ly/3dDWXu1). The same sensor can also observe other kinds of phenomena such as temperature and air humidity. Battery level values are also recorded. To transfer the measurements captured, the sensor is coupled to a microcontroller with 3G connectivity. The node consists of open hardware and other elements such as 3D-printed pieces to join all the components. Six nodes were built and deployed in six classrooms in two different schools in two different periods. In the first school, located in Vilafamés (Castellón, Spain), a total of 38,891 observations were carried out. Altogether 34,570 measurements were captured in the second school located in Vall d'Alba (Castellón, Spain). Each node transmitted all the measurements to the main server in real time every 5 minutes. The resulting dataset has been published as raw data, which means that invalid or missing values may appear. These could have been caused by the low-quality sensors themselves, the lack of 3G coverage or other node deployment failures [2]. The raw data are provided in two different Comma-Separated Values (CSV) files, each of them contains the data for each school.

The following tables (Tables 1 and 2) examine summary statistics for each phenomenon and report the number of measurements, mean, standard deviation, median, minimum, maximum and range. Moreover, the tables also show some detailed summary statistics components such as trimmed (truncated) mean, median absolute deviation (Mad) and standard error (se). Skewness (skew) and kurtosis are also reported as a measure of symmetry or asymmetry of the data distribution. To distinguish which node has generated the observations, a sensor identifier has been used.

**Table 1:** Summary of the temperature, relative humidity and CO_2_. CEIP Sant Miquel, Vilafamés, Castellón, Spain.

	N	Mean	Sd	Median	Trimmed	Mad	Min	Max	Range	skew	Kurtosis	se
**sensor_id: CO2_01**												
CO_2_	6670	462.56	100.69	428	441.68	44.48	353	1133	780	2.45	7.12	1.23
temp	6670	27.32	1.27	27.4	27.36	1.19	23.9	31	7.1	–0.29	–0.33	0.02
hum	6670	39.99	4.9	40.6	40.46	4.69	21.2	54.4	33.2	–0.86	0.41	0.06

**sensor_id: CO2_02**												
CO_2_	6925	472.78	78.65	450	457.89	37.06	377	1213	836	2.7	11.02	0.95
temp	6925	26.87	1.56	26.9	26.86	1.78	20.8	30.7	9.9	–0.06	–0.7	0.02
hum	6925	41.57	4.69	42.8	42.09	2.67	20.4	52.9	32.5	–1.23	1.96	0.06

**sensor_id: CO2_03**												
CO_2_	4739	443.37	53.69	428	434.97	35.58	367	745	378	1.66	3.18	0.78
temp	4739	26.51	1.91	27	26.65	1.63	20.7	30.8	10.1	–0.61	–0.24	0.03
hum	4739	40.18	6.24	41.4	40.81	4.89	17.1	61.54	44.44	–1.03	1.17	0.09

**sensor_id: CO2_04**												
CO_2_	7552	471.93	72.27	448	459.35	47.44	376	941	565	1.81	3.95	0.83
temp	7552	24.26	1.93	24.1	24.12	1.48	20	39.9	19.9	2.2	11.98	0.02
hum	7552	45.72	7.05	47.57	46.56	6.42	14.3	59.2	44.9	–1.06	0.86	0.08

**sensor_id: CO2_05**												
CO_2_	6162	462.42	60.33	444	451.5	29.65	384	998	614	2.71	11.29	0.77
temp	6162	26.56	1.32	26.6	26.59	1.33	21	30.6	9.6	–0.21	0.1	0.02
hum	6162	41.84	5.53	42.9	42.54	4.6	20.6	63.9	43.3	–1.16	1.32	0.07

**sensor_id: CO2_06**												
CO_2_	6842	452.44	65.89	442	445.65	41.51	301	1135	834	1.56	5.28	0.8
temp	6842	26.97	1.65	27	27.03	1.48	20.2	31.8	11.6	–0.27	0.15	0.02
hum	6842	40.02	5.69	40.8	40.57	5.63	18.9	60.66	41.76	–0.83	0.55	0.07

**Table 2:** Summary of the temperature, relative humidity and CO_2_. CEIP L'Albea, Vall d'Alba, Castellón, Spain.

	N	Mean	Sd	Median	Trimmed	Mad	Min	Max	Range	skew	Kurtosis	Se
**sensor_id: CO2_01**												
CO_2_	6201	443.14	88.73	417.00	421.00	14.83	386.00	1277.00	891.00	4.04	18.56	1.13
temp	6201	28.93	1.43	29.11	28.95	1.48	24.83	32.23	7.40	–0.16	–0.74	0.02
hum	6201	45.79	6.95	46.67	46.21	7.52	25.18	57.94	32.76	–0.46	–0.52	0.09

**sensor_id: CO2_02**												
CO_2_	6132	490.62	186.98	440.00	445.71	40.03	358.0	1989.00	1631.00	3.97	18.26	2.39
temp	6132	29.63	1.53	29.78	29.58	1.68	22.6	39.55	16.95	0.33	0.37	0.02
hum	6132	46.60	6.02	48.07	46.99	6.26	25.5	64.18	38.68	–0.58	–0.20	0.08

**sensor_id: CO2_03**												
CO_2_	6246	456.74	131.92	420.00	424.04	16.31	386.00	1961.00	1575.00	4.74	27.55	1.67
temp	6246	31.18	1.44	31.23	31.15	1.59	25.26	35.10	9.84	0.13	–0.61	0.02
hum	6246	40.62	6.16	42.36	41.12	6.21	21.12	55.96	34.84	–0.65	–0.31	0.08

**sensor_id: CO2_04**												
CO_2_	4239	495.52	193.42	433.00	442.17	26.69	381.00	1723.00	1342.00	3.22	10.67	2.97
temp	4239	27.46	1.38	27.08	27.26	1.11	23.72	31.36	7.64	1.15	0.71	0.02
hum	4239	49.47	6.72	50.69	50.19	6.60	26.77	62.30	35.53	–0.88	0.34	0.10

**sensor_id: CO2_05**												
CO_2_	5607	450.24	85.55	427.00	428.37	14.83	363.00	1189.00	826.00	3.81	16.16	1.14
temp	5607	30.92	1.86	30.72	30.74	1.45	24.64	44.46	19.82	2.39	11.38	0.02
hum	5607	43.92	5.63	45.04	44.50	4.23	21.04	57.53	36.49	–1.03	1.00	0.08

**sensor_id: CO2_06**												
CO_2_	6143	461.39	79.08	441.00	442.46	17.79	389.00	1044.00	655.00	3.63	14.16	1.01
temp	6143	30.93	1.48	30.97	30.90	1.69	25.13	35.23	10.10	0.18	–0.53	0.02
hum	6143	42.62	6.10	44.59	43.35	4.08	21.30	55.48	34.18	–1.10	0.73	0.08

The dataset depicted in the present study was used to explore spatiotemporal variations in indoor air quality in secondary schools in urban areas of Spain [3]. We analysed spatial and temporal variations of CO_2_ in classrooms using other atmospheric covariates such as temperature and humidity. Moreover, the study was conducted to identify the variation in CO_2_ concentrations based on the number of students, class hours, schedule and duration of breaks between classes, and the size and location of classrooms. The study can provide strategic support for administration and researchers on adequate ventilation of indoor public places.

There has been extensive evidence showing that prolonged exposure to high levels of CO_2_ concentration is detrimental to the performance of schoolchildren [4,5]. Hence, a second study was conducted to quantitatively address similar previously suspected problems based on some sparse dataset. The study was designed to test how CO_2_ concentration in classrooms influences the level of student attention and reduces mental performance. Observations of CO_2_ concentrations could be extended in different classrooms throughout the entire academic year to improve the study. Further development of the study can be performed in other schools with varying types of ventilation during the same time frame.

## 2 Experimental Design, Materials and Methods

### 2.1 Hardware components and materials

**Fig. 1:**
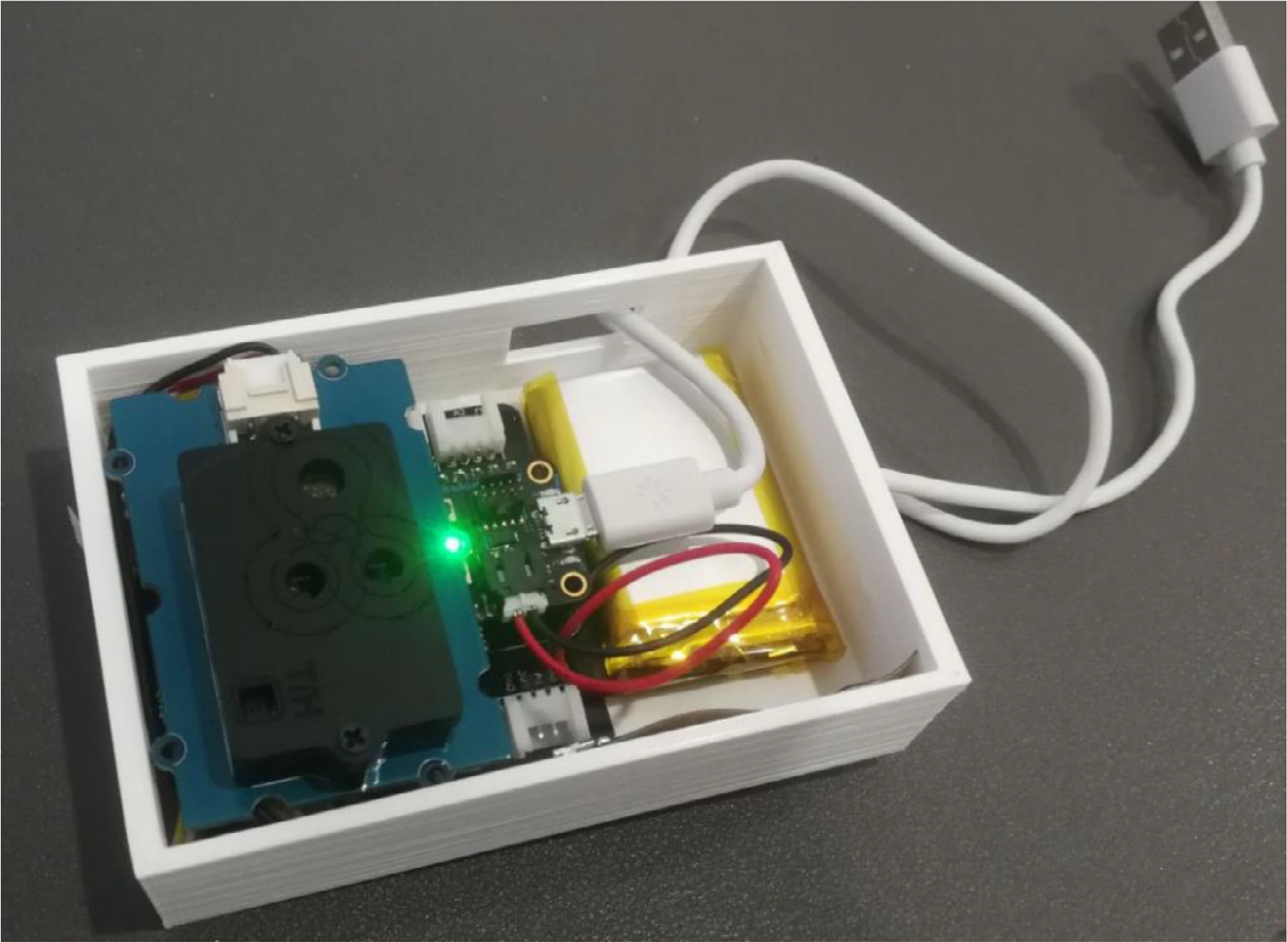
Fully assembled and deployed CO2, temperature and air humidity node.

The published data were collected using a set of nodes created by the authors. These nodes were based on the nodes presented in [6,7] and were built using open-hardware components (Fig. 1). The cited structure defined four different groups Core, Sensing/Acting, Power Supply and Communication. In the following, each category is defined by summarising each component used to build a node:

• **Core**. The core is the main part and is responsible for managing all the behaviour of the IoT node. The microcontroller used is the Particle Boron and follows an open-source design. It includes the Nordic nRF52840 (ARM Cortex-M4F 32-bit processor @ 64MHz and 1MB flash, 256KB RAM) and u-blox SARA U201 (2G/3G), with built-in battery charging circuitry, which makes it easier to connect a Li-Po battery, and 20 mixed-signal GPIOs to interface with sensors, actuators and other electronics.
• **Sensing/Acting**. A shield circuit compatible with the Particle Boron microcontroller is used. This component can connect and disconnect the sensors and actuators easily. Only one sensor is included, the SCD30 from the Sensirion company. It can measure CO_2_, temperature and humidity. The sensor supports CMOSens technology for IR detection that enables highly accurate carbon dioxide measurement at a competitive price.
• **Power supply**. The node is connected using a USB cable to provide power. An 800 mAh Li-po battery is included in the node to offer an autonomous solution in case of any energy supply issues.
• **Communication**. As previously mentioned, this node offers 3G communication using a SIM card.

Each phenomenon collected is described below, providing details of units, range and accuracy.

• **CO_2_**. Manufacturer: *Sensirion*; Model: *SCD30*; Data Interface: digital interface (*I2C/UART)*; Units: *ppm*; Range: *[0, 40000]*; Accuracy: *±30 ppm*
• **Temperature**. Manufacturer: *Sensirion*; Model: *SCD30*; Data Interface: digital interface (*I2C/UART)*; Units: *Centigrade*; Range: *[-40, 70]*; Accuracy: *±0.4° (C)*
• **Humidity**. Manufacturer: *Sensirion*; Model: *SCD30*; Data Interface: digital interface (*I2C/UART)*; Units: *%RH*; Range: *[0%, 100%]*; Accuracy: *±3 RH*

### 2.2 Deployment

**Fig. 2:**
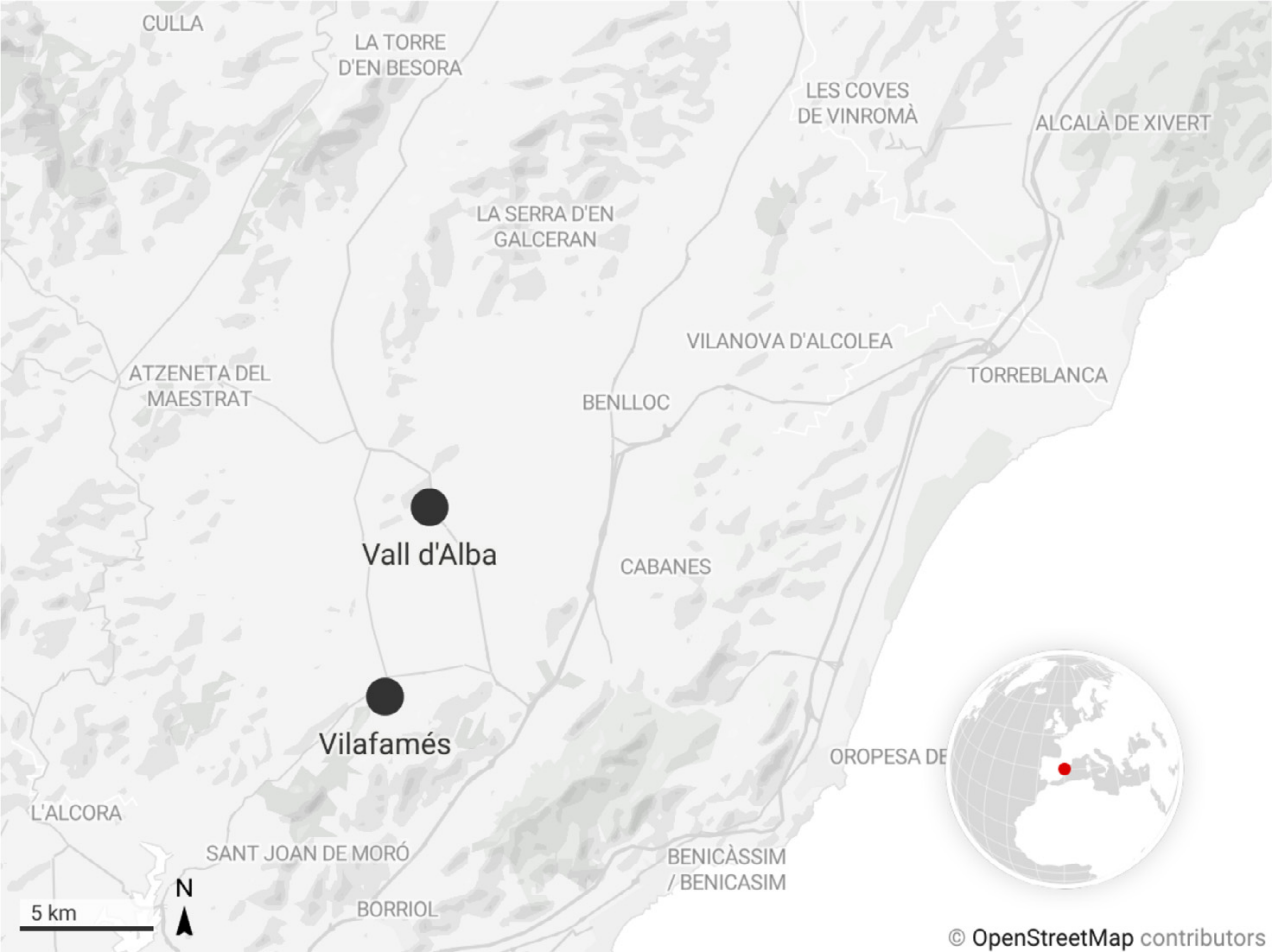
Map showing the locations of each of the schools.

Six units of the node were built and all of them were placed in different classrooms in two different schools during two different periods (Fig. 2). The first school (CEIP Sant Miquel) is located in Vilafamés (40.1162389, -0.0467876). The total population of schoolchildren in CEIP Sant Miquel is 154, with 101 pupils in primary education and 53 in preschool.

The second school is located close to Vilafamés, in Vall d'Alba (40.1763202, -0.0387236). This school is called CEIP L'Albea, and the total population of schoolchildren is 272, with 186 attending primary school and 86 in preschool.

In the Vilafamés school, the sensors collected observations during the period 1 May 2020 to 28 May 2020, and in the CEIP L'Albea during the period 1 June 2021 to 23 June 2021.

**Fig. 3:**
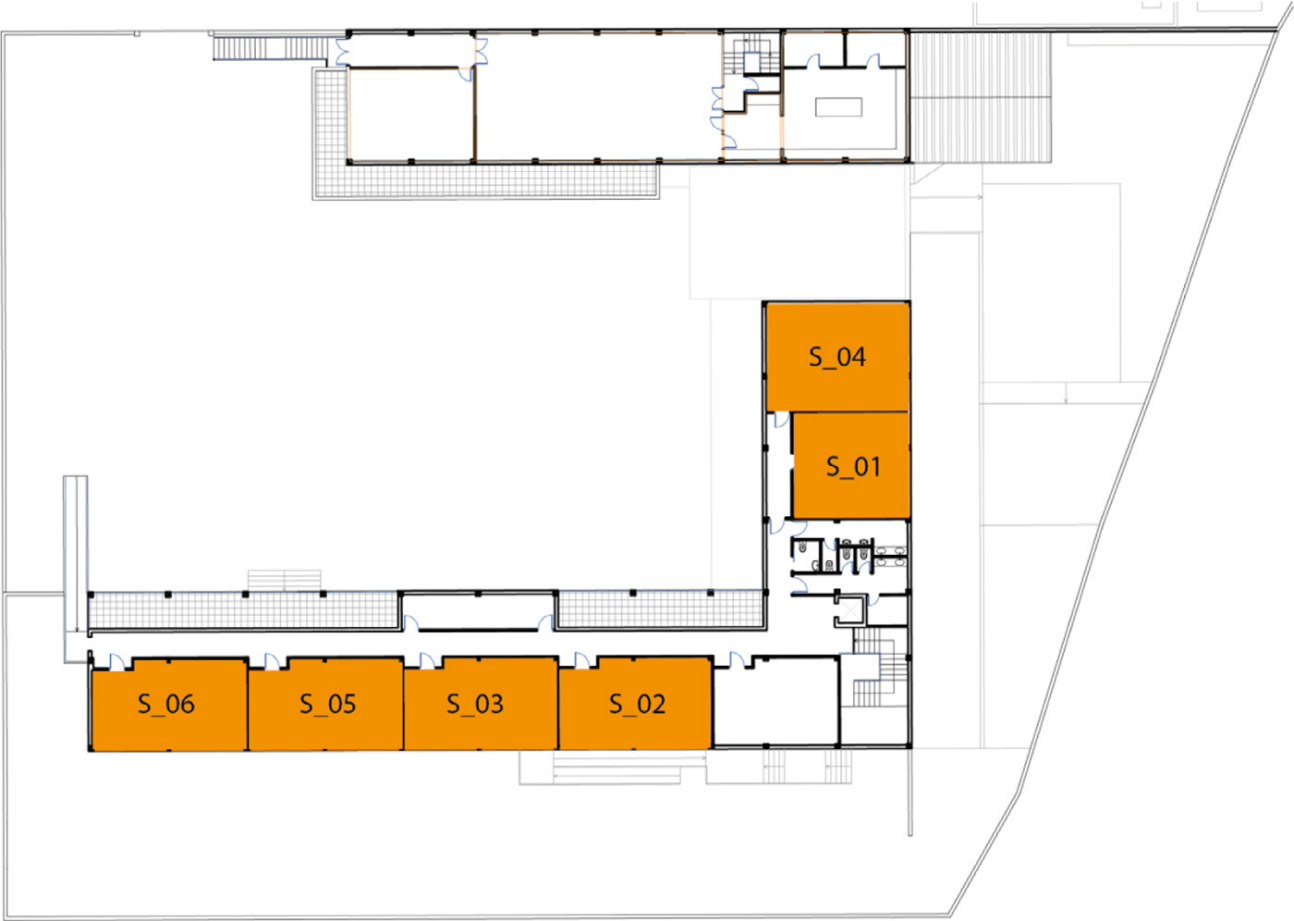
Plan of the Vilafamés school (CEIP Sant Miquel).

Fig. 3 shows the plans of CEIP Sant Miquel, the classes where each sensor was installed being indicated in orange. In addition, each of them has been labelled with the sensor identifier. Table 3 shows the characteristics of each of the classrooms where a sensor was installed, indicating the level of education, the number of pupils, the square metres of each space and the number of windows and doors. Table 4 shows the timetables when each classroom was in use by the students. Table 5 shows the different activities carried out outside the classroom by each class during the monitoring period.

**Table 3:** Summary of the features of the CEIP Sant Miquel (Vilafamés) classrooms.

*Sensor Id*	Grade	Number of students	Metres	Windows	Doors	Orientation of classrooms
S_01	2nd	16	39.56	3	1	Playground
S_02	6th	18	43.37	4	1	Street
S_03	4th	15	43.37	4	1	Street
S_04	1st	17	49.61	6	1	Playground
S_05	5th	18	43.37	4	1	Street
S_06	3rd	17	43.37	4	1	Street

**Table 4:** Summary of student attendance schedules in each of the classrooms during the monitoring period at the Vilafamés school.

*Sensor Id (classroom)*	Monday	Tuesday	Wednesday	Thursday	Friday
S_01 (2nd)	9.30-11.00	10.15-11.00	9.30-11.00	9.30-11.00	11.30-13.00
	11.30-13.00	11.30-13.00	11.30-13.00	15.30-17.00	15.30-17.00
	15.30-17.00	15.30-17.00	15.30-17.00		

S_02 (6th)	9.30-11.45	9.30-11.45	11.00-11.45	9.30-11.45	9.30-11.45
	12.15-13.00	15.30-17.00	12.15-13.00	12.15-13.00	12.15-13.00
	15.30-17.00		15.30-17.00	15.30-17.00	15.30-17.00

S_03 (4th)	9.30-11.45	9.30-11.45	9.30-11.45	9.30-11.45	9.30-11.45
	15.30-17.00	12.15-13.00	12.15-13.00	12.15-13.00	12.15-13.00
		15.30-17.00	15.30-17.00		15.30-17.00

S_04 (1st)	9.30-11.00	9.30-10.15	9.30-11.00	9.30-11.00	9.30-11.00
	11.30-13.00	15.30-17.00	11.30-13.00	11.30-13.00	11.30-13.00
	15.30-17.00		15.30-17.00	15.30-17.00	

S_05 (5th)	10.15-11.45	11.00-11.45	11.00-11.45	9.30-11.45	9.30-11.45
	12.15-13.00	12.15-13.00	12.15-13.00	12.15-13.00	12.15-13.00
	15.30-17.00	15.30-17.00	15.30-17.00	15.30-17.00	15.30-17.00

S_06 (3rd)	9.30-11.45	9.30-11.45	9.30-11.45	9.30-11.45	9.30-11.45
	12.15-13.00	15.30-17.00	12.15-13.00	12.15-13.00	12.15-13.00
	15.30-17.00			15.30-17.00	15.30-17.00

**Table 5:** Summary of the activities carried out outside the usual hours of attendance during the monitoring period at the Vilafamés school.

*Sensor Id (classroom)*	Day	Hours
S_01 (2nd)	14/05/2021	13.00 to 17.00
S_02 (6th)	20/05/2021	9.30 to 17.00
S_03 (4th)	13/05/2021	13.00 to 17.00
S_04 (1st)	07/05/2021	13.00 to 17.00
S_05 (5th)	03/05/2021	9.30 to 13.00
S_06 (3rd)	12/05/2021	13.00 to 17.00

**Fig. 4:**
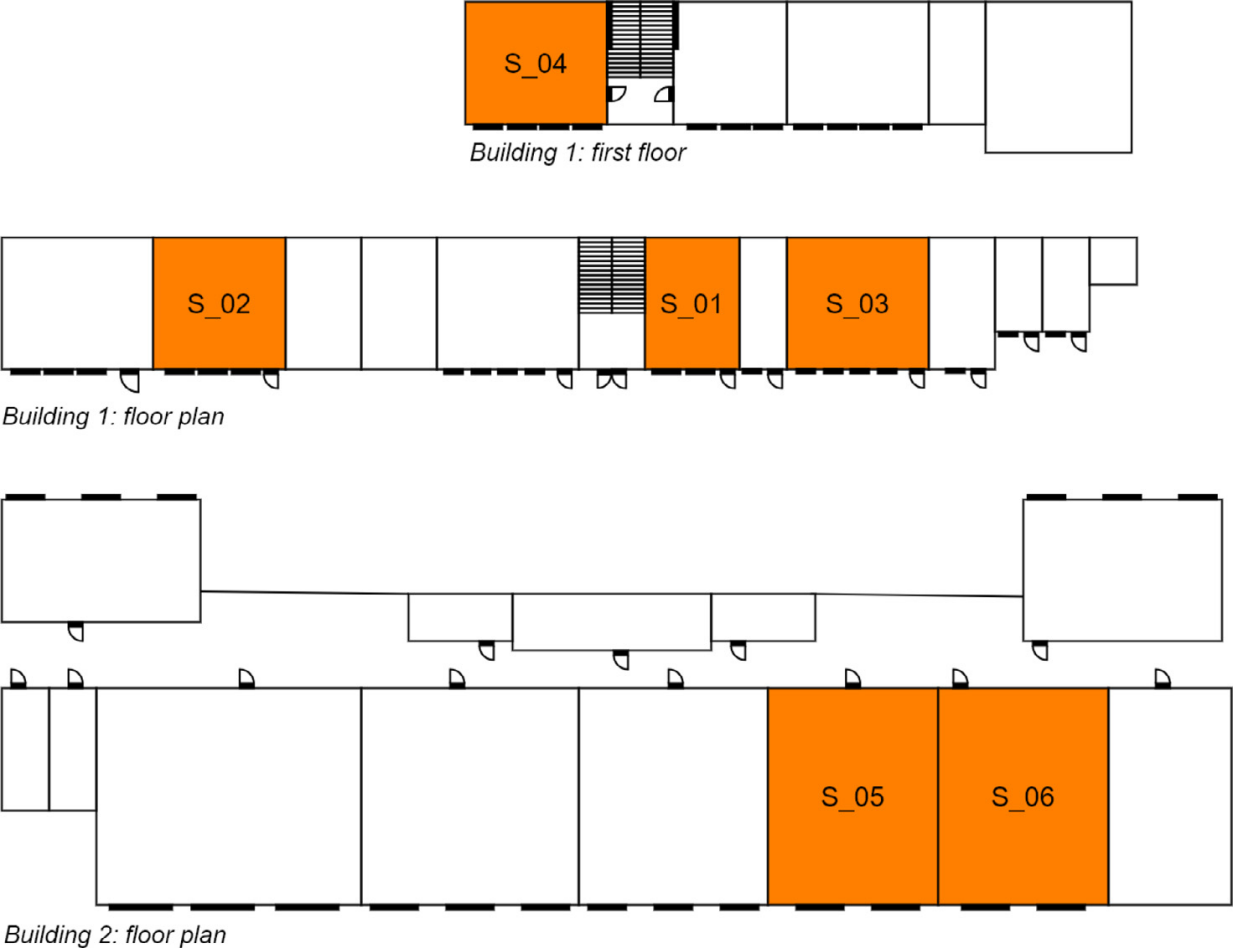
Plan of the Vall d'Alba school (CEIP L'Albea).

Below, Fig. 4 and Table 6, Table 7, Table 8 show the same information for the Vall d'Alba school.

**Table 6:** Summary of the features of the CEIP L'Albea (Vall d'Alba) classrooms.

*Sensor Id*	Grade	Number of students	Metres	Windows	Doors	Orientation of classrooms
S_01	1r	16	35.8	2	1	Playground
S_02	6e	20	65.3	3	1	Playground
S_03	4t	16	67	4	1	Playground
S_04	5e	14	69.17	4	1	Playground
S_05	3r	24	44.56	2	1	Playground
S_06	2n	25	40.9	2	1	Playground

**Table 7:** Summary of student attendance schedules in each of the classrooms during the monitoring period at the Vall d'Alba school.

*Sensor Id*	Monday	Tuesday	Wednesday	Thursday	Friday
S_01 (1st)	9.45-11.00, 11.30-13.00	9.30-11.00, 11.30-13.00	9.30-11.00, 11.30-13.00	9.30-11.00, 11.30-13.00	9.30-11.00, 11.30-12.00
S_02 (6th)	9.00-11.00, 12.15-13.00	10.00-11.00, 11.30-13.00	9.00-11.00, 11.30-13.00	9.00-11.00, 11.30-13.00	9.00-11.00, 11.30-13.00
S_03 (4th)	9.00-11.00, 11.30-13.00	9.00-11.00, 11.30-12.15	9.00-10.00, 11.30-13.00	9.00-11.00, 11.30-13.00	9.00-11.00, 11.30-13.00
S_04 (5th)	9.00-11.00, 11.30-13.00	9.00-11.00, 11.30-13.00	9.00-11.00, 11.30-12.15	9.00-10.00, 11.30-13.00	9.00-11.00, 11.30-13.00
S_05 (3rd)	9.00-11.00, 12.15-13.00	9.00-11.00, 11.30-13.00	9.00-11.00, 11.30-13.00	9.00-11.00, 12.15-13.30	9.45-11.00, 11.00-13.30
S_06 (2nd)	9.00-11.00, 11.30-13.00	9.00-11.00, 11.30-13.00	9.00-11.00, 11.30-13.00	9.00-10.00, 11.30-12.15	9.45-11.00, 11.30-13.00

**Table 8:** Summary of the activities carried out outside the usual hours of attendance during the monitoring period at the Vall d'Alba school.

*Sensor Id (classroom)*	Day	Hours	*Sensor Id (classroom)*	Day	Hours
S_01 (2nd)	15/06/2021	9.15 to 10.15	S_04 (1st)	14/06/2021	9.15 to 10.15
	16/06/2021	11.00 to 12.00		16/06/2021	11.00 to 12.00
	17/06/2021	11.00 to 12.00		18/06/2021	9.30 to 10.30
	21/06/2021	11.00 to 12.00		21/06/2021	11.00 to 12.00
	21/06/2021	12.30 to 13.00		21/06/2021	12.30 to 13.00
	22/06/2021	9.30 to 10.30		22/06/2021	9.30 to 10.30
	22/06/2021	12.00 to 13.00		22/06/2021	12.00 to 13.00
	23/06/2021	9.00 to 13.00		23/06/2021	9.00 to 13.00

S_02 (6th)	15/06/2021	12.00 to 13.00	S_05 (5th)	14/06/2021	12.00 to 13.00
	17/06/2021	9.00 to 10.00		15/06/2021	11.30 to 12.15
	18/06/2021	11.40 to 12.20		18/06/2021	10.20 to 11.00
	21/06/2021	9.15 to 11.00		21/06/2021	9.15 to 11.00
	21/06/2021	12.30 to 13.00		21/06/2021	12.30 to 13.00
	22/06/2021	9.30 to 10.00		22/06/2021	9.30 to 10.30
	22/06/2021	11.00 to 12.00		22/06/2021	11.00 to 12.00
	23/06/2021	9.00 to 13.00		23/06/2021	9.00 to 13.00

S_03 (4th)	14/06/2021	10.15 to 11.00	S_06 (3rd)	15/06/2021	9.15 to 10.15
	16/06/2021	9.00 to 10.00		16/06/2021	11.00 to 12.00
	18/06/2021	9.00 to 9.45		17/06/2021	12.00 to 13.00
	21/06/2021	9.15 to 11.00		21/06/2021	11.00 to 12.00
	21/06/2021	12.30 to 13.00		21/06/2021	12.30 to 13.00
	22/06/2021	9.30 to 10.30		22/06/2021	9.30 to 10.30
	22/06/2021	11.00 to 12.00		22/06/2021	12.00 to 13.00
	23/06/2021	9.00 to 13.00		23/06/2021	9.00 to 13.00

## Particle Sketches (code)

The source code developed to collect and send measurements to a main server using 3G connectivity is available in the following repository [8].

## Ethics Statement

The raw data of this study is provided by open in full compliance with ethical requirements for publication in the journal of Data in Brief. This study does not involve any modern human or animal subject.

## CRediT authorship contribution statement

**Sergio Trilles:** Supervision, Conceptualization, Methodology, Writing – original draft, Project administration, Funding acquisition, Writing – original draft. **Pablo Juan:** Software, Data curation, Writing – review & editing. **Somnath Chaudhuri:** Formal analysis, Validation, Writing – original draft. **Ana Belen Vicente Fortea:** Writing – review & editing.

## Declaration of Competing Interest

The authors declare that they have no known competing financial interests or personal relationships which have, or could be perceived to have, influenced the work reported in this article.
